# The genome sequence of the yellow-tail moth, *Euproctis similis* (Fuessly, 1775)

**DOI:** 10.12688/wellcomeopenres.17188.2

**Published:** 2021-12-08

**Authors:** Douglas H. Boyes, Peter W.H. Holland, University of Oxford and Wytham Woods Genome Acquisition Lab, Darwin Tree of Life Barcoding collective, Wellcome Sanger Institute Tree of Life programme, Wellcome Sanger Institute Scientific Operations: DNA Pipelines collective, Tree of Life Core Informatics collective, Darwin Tree of Life Consortium

**Affiliations:** 1 UK Centre for Ecology & Hydrology, Wallingford, Oxfordshire, OX10 8BB, UK; 2 Department of Zoology, University of Oxford, Oxford, OX1 3SZ, UK

**Keywords:** Euproctis similis, yellow-tail, genome sequence, chromosomal

## Abstract

We present a genome assembly from an individual male *Euproctis similis *(the yellow-tail; Arthropoda; Insecta; Lepidoptera; Lymantriidae). The genome sequence is 508 megabases in span. Over 99% of the assembly is scaffolded into 22 chromosomal pseudomolecules, with the Z sex chromosome assembled. The complete mitochondrial genome, 15.5 kb in length, was also assembled.

## Species taxonomy

Eukaryota; Metazoa; Ecdysozoa; Arthropoda; Hexapoda; Insecta; Pterygota; Neoptera; Endopterygota; Lepidoptera; Glossata; Ditrysia; Noctuoidea; Erebidae; Lymantriinae; Euproctis; *Euproctis similis* Fuessly 1775 (NCBI:txid987935).

## Introduction

*Euproctis similis*, the yellow-tail moth, is widespread across temperate Europe and Asia. In the UK, the moth is relatively common across much of England and Wales, with scattered records from southern Scotland and Northern Ireland. The larvae of *E. similis* feed on a range of deciduous trees and shrubs, including *Crataegus, Prunus*, and *Betula*, in some situations becoming a pest on ornamental and fruit trees. Larvae of are also notable for bearing long hairs that can cause skin irritation in humans, although the effects are rarely as serious as those caused by larvae of the closely related *Euproctis chrysorrhoea* (brown-tail). A genome sequence for *E. similis*, therefore, may have agricultural and biomedical relevance, in addition to its use in evolutionary biology, ecology and genome biology. The karyotype of E. similis has been previously recorded as n=22 or 23 ( Belyakova & Lukhtanov, 1994). This is not unexpected since Lepidoptera exhibit considerable variation in chromosome number, although n=31 is the most common karyotype ( Ahola *et al.*, 2014). The genome of *E. similis* was sequenced as part of the Darwin Tree of Life Project, a collaborative effort to sequence all of the named eukaryotic species in the Atlantic Archipelago of Britain and Ireland. Here we present a chromosomally complete genome sequence for *E. similis*, based on one male specimen from Wytham Woods, Oxfordshire (biological vice-country: Berkshire), UK.

## Genome sequence report

**Figure 1.**
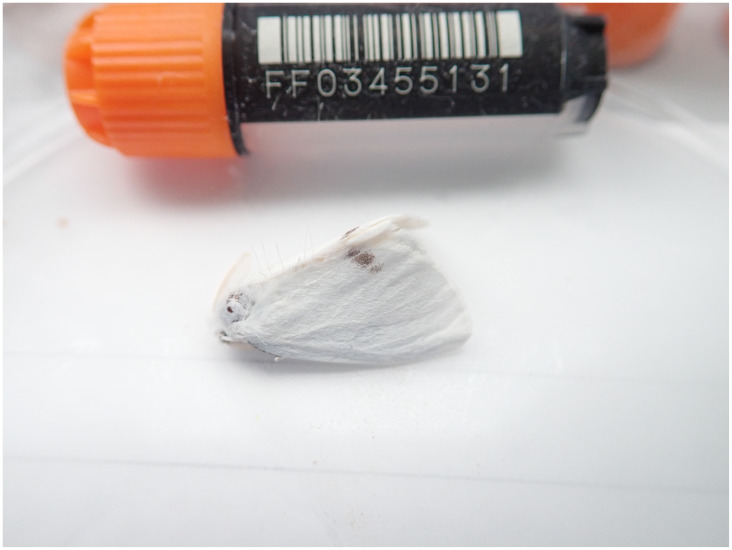
Image of the *Euproctis similis* specimen (ilEupSimi1) used for genome sequencing. Image captured during preservation and processing. Specimen is shown below a FluidX storage tube 43.9 mm in length.

**Figure 2.**
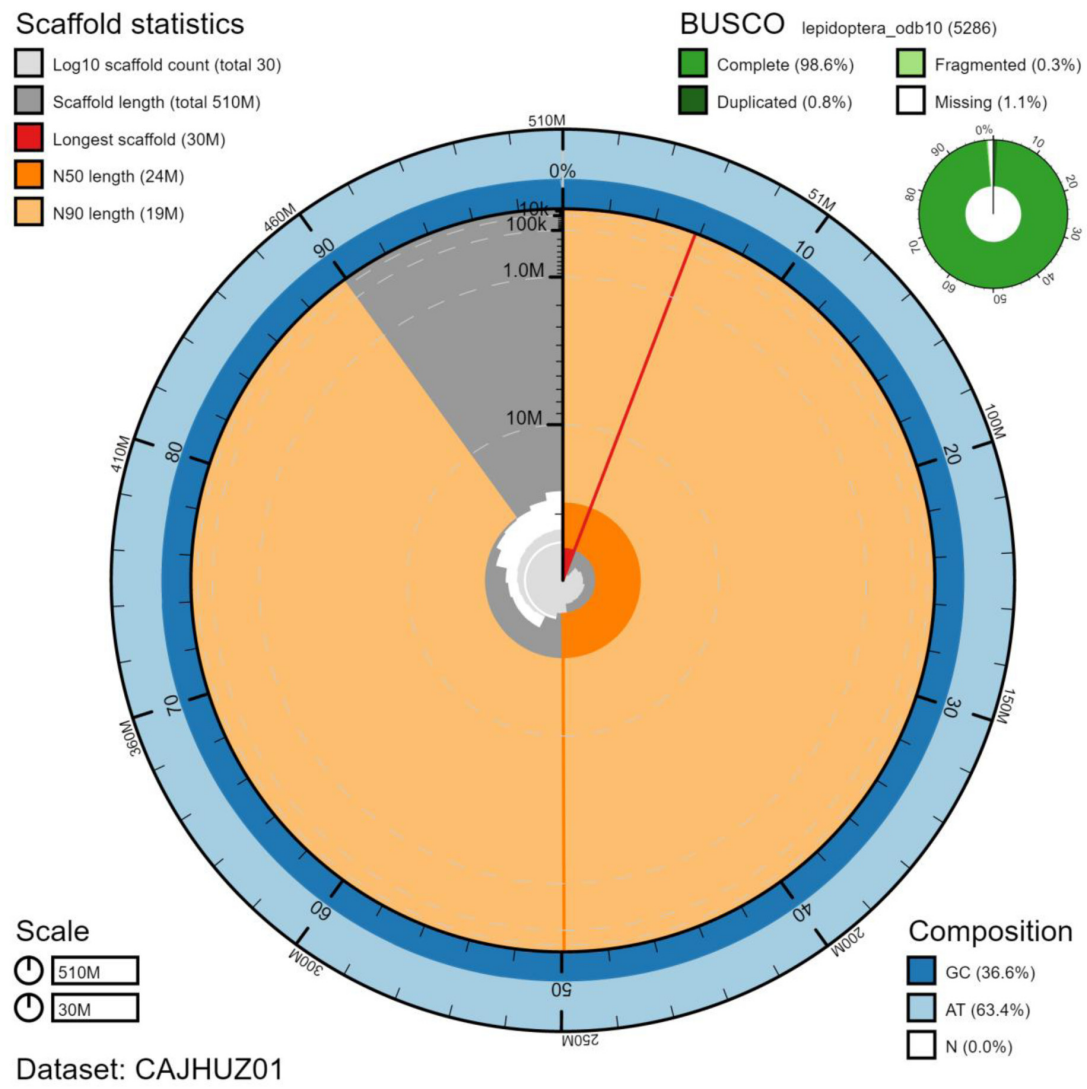
Genome assembly of *Euproctis similis*, ilEupSimi1.1: metrics. The BlobToolKit Snailplot shows N50 metrics and BUSCO gene completeness. The main plot is divided into 1,000 size-ordered bins around the circumference with each bin representing 0.1% of the 507,648,621 bp assembly. The distribution of scaffold lengths is shown in dark grey with the plot radius scaled to the longest scaffold present in the assembly (29,627,438 bp, shown in red). Orange and pale-orange arcs show the N50 and N90 scaffold lengths (24,449,160 and 18,514,210 bp), respectively. The pale grey spiral shows the cumulative scaffold count on a log scale with white scale lines showing successive orders of magnitude. The blue and pale-blue area around the outside of the plot shows the distribution of GC, AT and N percentages in the same bins as the inner plot. A summary of complete, fragmented, duplicated and missing BUSCO genes in the lepidoptera_odb10 set is shown in the top right.An interactive version of this figure is available at https://blobtoolkit.genomehubs.org/view/ilEupSimi1.1/dataset/CAJHUZ01/snail.

The genome was sequenced from a single male *E. similis* ( Figure 1) collected from Wytham Woods, Oxfordshire (biological vice-county: Berkshire), UK (latitude 51.772, longitude -1.338). A total of 70-fold coverage in Pacific Biosciences single-molecule long reads (N50 17 kb) and 78-fold coverage in 10X Genomics read clouds were generated. Primary assembly contigs were scaffolded with chromosome conformation Hi-C data. Manual assembly curation corrected 40 missing/misjoins and removed 3 haplotypic duplications, reducing the assembly length by 0.10% and the scaffold number by 42.00%, and increasing the scaffold N50 by 14.24%. The final assembly has a total length of 508 Mb in 30 sequence scaffolds with a scaffold N50 of 24 Mb ( Table 1). Over 99.9% of the assembly sequence was assigned to 22 chromosomal-level scaffolds, representing 21 autosomes (numbered by sequence length), and the Z sex chromosome ( Figure 2– Figure 5; Table 2). The assembly has a BUSCO ( Simão *et al*., 2015) v5.1.2 completeness of 98.6% using the lepidoptera_odb10 reference set. The complete, unbroken mitochondrial genome was assembled and is 15.5 kb in length. While not fully phased, the assembly deposited is of one haplotype. Contigs corresponding to the second haplotype have also been deposited.

**Table 1.** Genome data for *Euproctis similis*, ilEupSimi1.1.

*Project accession data*	
Assembly identifier	ilEupSimi1.1
Species	*Euproctis similis*
Specimen	ilEupSimi1
NCBI taxonomy ID	NCBI:txid987935
BioProject	PRJEB42127
BioSample ID	SAMEA7519909
Isolate information	Male, head/abdomen/thorax
*Raw data accessions*	
PacificBiosciences SEQUEL II	ERR6406199
10X Genomics Illumina	ERR6002639-ERR6002642
Hi-C Illumina	ERR6002643, ERR6002644
Illumina PolyA RNAseq	ERR6286704
*Genome assembly*	
Assembly accession	GCA_905147225.1
*Accession of alternate haplotype*	GCA_905147215.1
Span (Mb)	508
Number of contigs	55
Contig N50 length (Mb)	21
Number of scaffolds	30
Scaffold N50 length (Mb)	24
Longest scaffold (Mb)	30
BUSCO * genome score	C:98.6%[S:97.7%,D:0.8%], F:0.3%,M:1.1%,n:5286

*BUSCO scores based on the lepidoptera_odb10 BUSCO set using v5.1.2. C= complete [S= single copy, D=duplicated], F=fragmented, M=missing, n=number of orthologues in comparison. A full set of BUSCO scores is available at https://blobtoolkit.genomehubs.org/view/ilEupSimi1.1/dataset/CAJHUZ01/busco.

**Figure 3.**
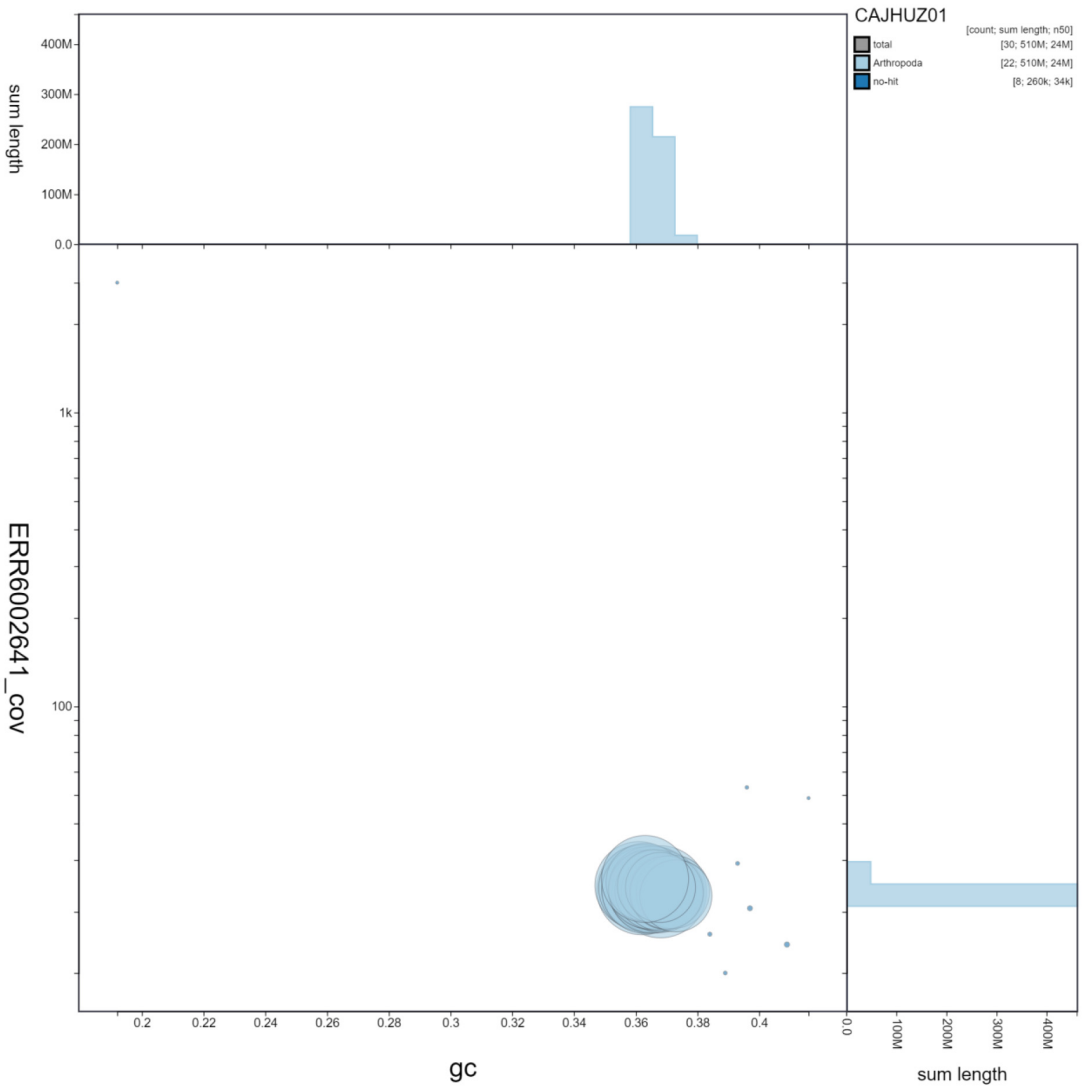
Genome assembly of *Euproctis similis*, ilEupSimi1.1: GC coverage. BlobToolKit GC-coverage plot. Scaffolds are coloured by phylum. Circles are sized in proportion to scaffold length. Histograms show the distribution of scaffold length sum along each axis. An interactive version of this figure is available at https://blobtoolkit.genomehubs.org/view/ilEupSimi1.1/dataset/CAJHUZ01/blob.

**Figure 4.**
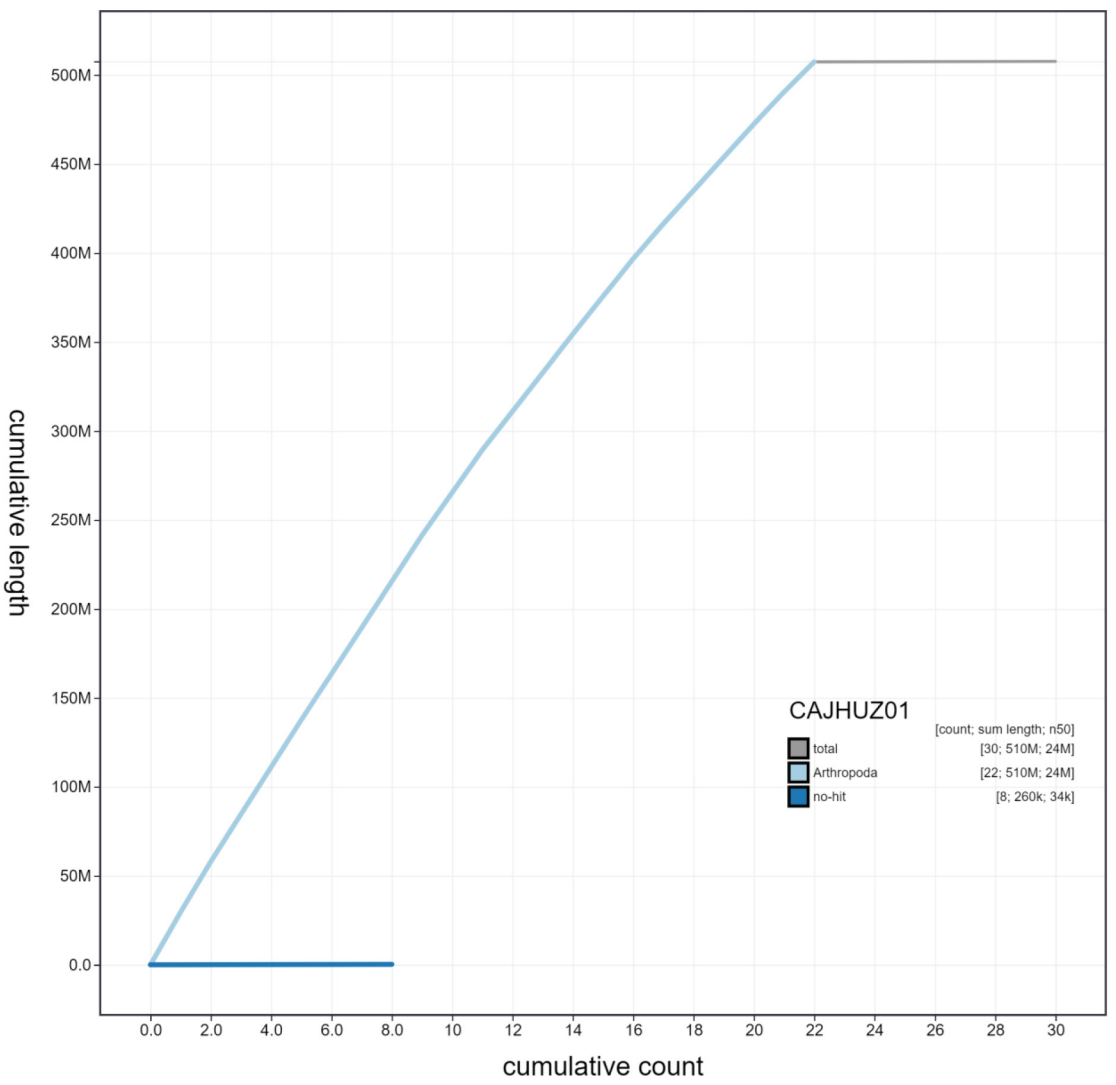
Genome assembly of *Euproctis similis*, ilEupSimi1.1: cumulative sequence. BlobToolKit cumulative sequence plot. The grey line shows cumulative length for all chromosomes. Coloured lines show cumulative lengths of chromosomes assigned to each phylum using the buscogenes taxrule. An interactive version of this figure is available at https://blobtoolkit.genomehubs.org/view/ilEupSimi1.1/dataset/CAJHUZ01/cumulative.

**Figure 5.**
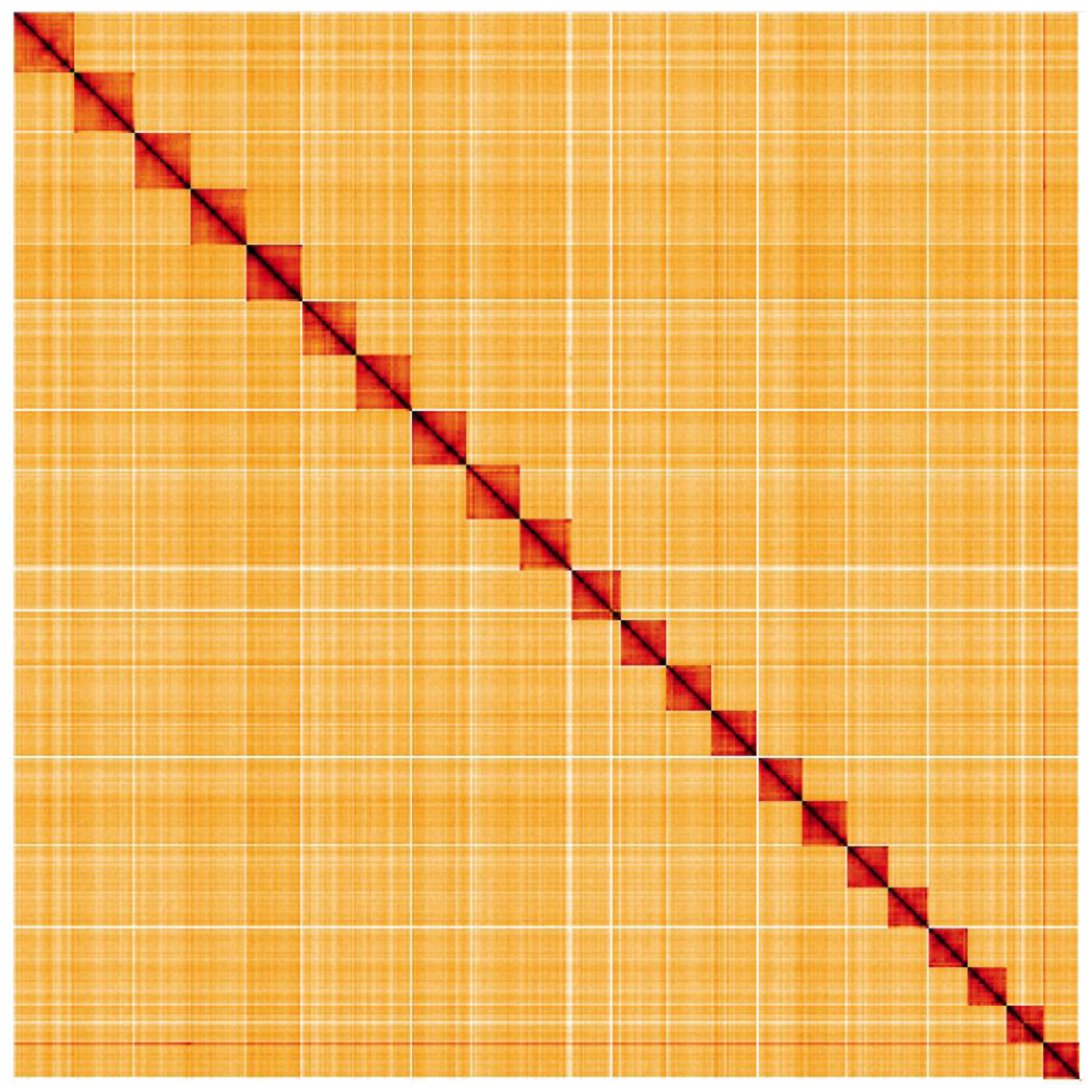
Genome assembly of *Euproctis similis*, ilEupSimi1.1: Hi-C contact map. Hi-C contact map of the ilEupSimi1.1 assembly, visualised in HiGlass. Chromosomes are arranged in size order, from left to right and top to bottom.

**Table 2.** Chromosomal pseudomolecules in the genome assembly of *Euproctis similis*, ilEupSimi1.1.

INSDC accession	Chromosome	Size (Mb)	GC%
LR990103.1	1	29.63	36.8
LR990104.1	2	28.43	36.2
LR990105.1	3	26.72	36.1
LR990106.1	4	26.40	36.3
LR990108.1	5	26.05	36.2
LR990109.1	6	26.03	36.3
LR990110.1	7	25.95	36.6
LR990111.1	8	25.82	36.5
LR990112.1	9	24.45	36.5
LR990113.1	10	23.51	36.8
LR990114.1	11	21.70	36.5
LR990115.1	12	21.66	36.4
LR990116.1	13	21.63	36.6
LR990117.1	14	21.40	36.8
LR990118.1	15	21.08	36.3
LR990119.1	16	19.59	37
LR990120.1	17	18.87	36.6
LR990121.1	18	18.67	37.2
LR990122.1	19	18.51	37
LR990123.1	20	17.96	37.3
LR990124.1	21	16.98	36.8
LR990107.1	Z	26.35	36.3
LR990125.1	MT	0.02	19.4
-	Unplaced	0.24	39.9

## Methods

A single male *E. similis*, ilEupSimi1, was collected from Wytham Woods, Oxfordshire (biological vice-country: Berkshire), UK (latitude 51.772, longitude -1.338) by Douglas Boyes, University of Oxford, using a light trap. The specimen was snap-frozen in dry ice using a CoolRack before transferring to the Wellcome Sanger Institute (WSI).

DNA was extracted at the Tree of Life laboratory, WSI. The ilEupSimi1 sample was weighed and dissected on dry ice with tissue set aside for RNA extraction and Hi-C sequencing. Thorax/abdomen tissue was cryogenically disrupted to a fine powder using a Covaris cryoPREP Automated Dry Pulveriser, receiving multiple impacts. Fragment size analysis of 0.01-0.5 ng of DNA was then performed using an Agilent FemtoPulse. High molecular weight (HMW) DNA was extracted using the Qiagen MagAttract HMW DNA extraction kit. Low molecular weight DNA was removed from a 200-ng aliquot of extracted DNA using 0.8X AMpure XP purification kit prior to 10X Chromium sequencing; a minimum of 50 ng DNA was submitted for 10X sequencing. HMW DNA was sheared into an average fragment size between 12-20 kb in a Megaruptor 3 system with speed setting 30. Sheared DNA was purified by solid-phase reversible immobilisation using AMPure PB beads with a 1.8X ratio of beads to sample to remove the shorter fragments and concentrate the DNA sample. The concentration of the sheared and purified DNA was assessed using a Nanodrop spectrophotometer and Qubit Fluorometer and Qubit dsDNA High Sensitivity Assay kit. Fragment size distribution was evaluated by running the sample on the FemtoPulse system.

RNA was extracted from thorax/abdomen tissue in the Tree of Life Laboratory at the WSI using TRIzol (Invitrogen), according to the manufacturer’s instructions. RNA was then eluted in 50 μl RNAse-free water and its concentration assessed using a Nanodrop spectrophotometer and Qubit Fluorometer using the Qubit RNA Broad-Range (BR) Assay kit. Analysis of the integrity of the RNA was done using Agilent RNA 6000 Pico Kit and Eukaryotic Total RNA assay.

Pacific Biosciences HiFi circular consensus and 10X Genomics Chromium read cloud sequencing libraries were constructed according to the manufacturers’ instructions. Poly(A) RNA-Seq libraries were constructed using the NEB Ultra II RNA Library Prep kit. Sequencing was performed by the Scientific Operations core at the Wellcome Sanger Institute on Pacific Biosciences SEQUEL II (HiFi), Illumina HiSeq X (10X) and Illumina HiSeq 4000 (RNA-Seq) instruments. Hi-C data were generated from head tissue using the Qiagen EpiTect Hi-C kit and sequenced on HiSeq X.

Assembly was carried out with HiCanu ( Nurk *et al*., 2020); haplotypic duplication was identified and removed with purge_dups ( Guan *et al*., 2020). The assembly was polished with the 10X Genomics Illumina data by aligning to the assembly with longranger align, calling variants with freebayes ( Garrison & Marth, 2012). One round of the Illumina polishing was applied. Scaffolding with Hi-C data ( Rao *et al*., 2014) was carried out with SALSA2 ( Ghurye *et al*., 2019). The assembly was checked for contamination and corrected using the gEVAL system ( Chow *et al*., 2016) as described previously ( Howe *et al*., 2021). Manual curation was performed using gEVAL, HiGlass ( Kerpedjiev *et al*., 2018) and Pretext. The mitochondrial genome was assembled using MitoHiFi ( Uliano-Silva *et al*., 2021). The genome was analysed and BUSCO scores generated within the BlobToolKit environment ( Challis *et al*., 2020). Table 3 contains a list of all software tool versions used, where appropriate.

**Table 3.** Software tools used.

Software tool	Version	Source
HiCanu	2.1	Nurk *et al*., 2020
purge_dups	1.2.3	Guan *et al*., 2020
SALSA2	2.2	Ghurye *et al*., 2019
longranger align	2.2.2	https:// support.10xgenomics.com/ genome-exome/software/ pipelines/latest/advanced/ other-pipelines
freebayes	1.3.1-17-gaa2ace8	Garrison & Marth, 2012
MitoHiFi	1	Uliano-Silva *et al*., 2021
gEVAL	N/A	Chow *et al*., 2016
HiGlass	1.11.6	Kerpedjiev *et al*., 2018
PretextView	0.1.x	https://github.com/wtsi- hpag/PretextView
BlobToolKit	2.6.2	Challis *et al*., 2020

The materials that have contributed to this genome note have been supplied by a Darwin Tree of Life Partner. The submission of materials by a Darwin Tree of Life Partner is subject to the Darwin Tree of Life Project Sampling Code of Practice. By agreeing with and signing up to the Sampling Code of Practice, the Darwin Tree of Life Partner agrees they will meet the legal and ethical requirements and standards set out within this document in respect of all samples acquired for, and supplied to, the Darwin Tree of Life Project. Each transfer of samples is further undertaken according to a Research Collaboration Agreement or Material Transfer Agreement entered into by the Darwin Tree of Life Partner, Genome Research Limited (operating as the WSI), and in some circumstances other Darwin Tree of Life collaborators.

## Data availability

European Nucleotide Archive: Euproctis similis (yellow-tail). Accession number PRJEB42127: https://identifiers.org/ena.embl:PRJEB42127

The genome sequence is released openly for reuse. The *E. similis* genome sequencing initiative is part of the Darwin Tree of Life (DToL) project. All raw sequence data and the assembly have been deposited in INSDC databases. The genome will be annotated using RNAseq data and presented through the Ensembl pipeline at the European Bioinformatics Institute. Raw data and assembly accession identifiers are reported in Table 1.
