# Supplementary material for: The genome sequence of the yellow-tail moth, Euproctis similis (Fuessly, 1775)
Source: Wellcome Open Res. 2021 Dec 8;6:227. Originally published 2021 Sep 13. [Version 2] doi: 10.12688/wellcomeopenres.17188.2 (PMC8474102; doi:10.12688/wellcomeopenres.17188.2)
Supplement: Peer review history

# Peer review: Reviewer response for version 2

**DOI:** 10.21956/wellcomeopenres.19033.r47562

**Published:** 2021-12-13

**Authors:** Niclas Backström, Daria Shipilina

**Affiliations:** 1 Evolutionary Biology Program, Department of Ecology and Genetics (IEG), Uppsala University, Uppsala, Sweden; 2 Evolutionary Biology Program, Department of Ecology and Genetics, Uppsala University, Uppsala, Sweden

All comments from the previous version of the manuscript have been addressed. We have no further comments.

 Very minor observations that the authors may want to look at:

- Intro Line 4 "Larvae of are....".
- Intro Line 7 "The karyotype of E. similis ....." Latin name should be in italics.

Are sufficient details of methods and materials provided to allow replication by others?

Yes

Is the rationale for creating the dataset(s) clearly described?

Yes

Are the datasets clearly presented in a useable and accessible format?

Partly

Are the protocols appropriate and is the work technically sound?

Yes

Reviewer Expertise:

NA

We confirm that we have read this submission and believe that we have an appropriate level of expertise to confirm that it is of an acceptable scientific standard.

# Peer review: Reviewer response for version 2

**DOI:** 10.21956/wellcomeopenres.19033.r47561

**Published:** 2021-12-09

**Authors:** Thomas Blankers

**Affiliations:** 1 Institute for Biodiversity and Ecosystem Dynamics, University of Amsterdam, Amsterdam, The Netherlands

I have no further comments.

Are sufficient details of methods and materials provided to allow replication by others?

Partly

Is the rationale for creating the dataset(s) clearly described?

Yes

Are the datasets clearly presented in a useable and accessible format?

Yes

Are the protocols appropriate and is the work technically sound?

Yes

Reviewer Expertise:

Evolutionary genomics, currently working on crickets, moths, and C. elegans

I confirm that I have read this submission and believe that I have an appropriate level of expertise to confirm that it is of an acceptable scientific standard.

# Peer review: Reviewer response for version 2

**DOI:** 10.21956/wellcomeopenres.19033.r47560

**Published:** 2021-12-09

**Authors:** Steven M. Van Belleghem

**Affiliations:** 1 Department of Biology, University of Puerto Rico-Rio Piedras, San Juan, Puerto Rico

I am happy with the changes made.

Are sufficient details of methods and materials provided to allow replication by others?

Yes

Is the rationale for creating the dataset(s) clearly described?

Yes

Are the datasets clearly presented in a useable and accessible format?

Partly

Are the protocols appropriate and is the work technically sound?

Yes

Reviewer Expertise:

Genomics, functional genomics, insects

I confirm that I have read this submission and believe that I have an appropriate level of expertise to confirm that it is of an acceptable scientific standard.

# Peer review: Reviewer response for version 1

**DOI:** 10.21956/wellcomeopenres.18991.r45902

**Published:** 2021-09-24

**Authors:** Thomas Blankers

**Affiliations:** 1 Institute for Biodiversity and Ecosystem Dynamics, University of Amsterdam, Amsterdam, The Netherlands

This data note presents the genome assembly of *Euproctis similis *as part of the Darwin Tree of Life Project. The information about the molecular lab methods is sufficient and clear. The appropriate, protocols have been used, following recommendations of the manufacturers and sequencing platforms. The quality of the genome is high, owing to the use of complementary sequencing technologies that allow for contiguous assemblies.

 I only have three small suggestions for additional information, although I also see that none of these are commonly supplied in the notes coming from the Darwin Tree of Life project. First, as a biologist, I would be interested in knowing a little bit more about the organism. For example that it is a night-active moth, wide-spread across the Eurasian continent, that they're active from August to June and that they are associated with both urban and non-urban habitats and with several host plants. Second, in the presentation of the methods there are no details about the bioinformatic analyses beyond the programs that were used. It would be good to specify any deviation from default settings or even to have a brief summary of the commands used to perform the analyses. This could be done in a separate file archived along with the note or in a Table, possibly integrated in Table 3. Third, the data presentation can benefit from brief expansion of the results. The interactive figures are nice, because some explanation of what is shown can also be found at the corresponding blobtoolkit repository. However, there is no text accompanying these figures beyond a single sentence referencing the number of scaffolds  and citing figures 1 through 4. Some expansion of the genome assembly statistics seems desirable. And figure 4 could use a legend as well as axis labels with the chromosome numbers. Again, I do see that other examples of notes on genomes coming from this project also do not necessarily contain these additional pieces of information, so I guess it is up to the authors to decide whether that continuity matters more or whether the details are simply not necessary.

Are sufficient details of methods and materials provided to allow replication by others?

Partly

Is the rationale for creating the dataset(s) clearly described?

Yes

Are the datasets clearly presented in a useable and accessible format?

Yes

Are the protocols appropriate and is the work technically sound?

Yes

Reviewer Expertise:

Evolutionary genomics, currently working on crickets, moths, and C. elegans

I confirm that I have read this submission and believe that I have an appropriate level of expertise to confirm that it is of an acceptable scientific standard.

# Peer review: Reviewer response for version 1

**DOI:** 10.21956/wellcomeopenres.18991.r45899

**Published:** 2021-09-20

**Authors:** Niclas Backström, Daria Shipilina

**Affiliations:** 1 Evolutionary Biology Program, Department of Ecology and Genetics (IEG), Uppsala University, Uppsala, Sweden; 2 Evolutionary Biology Program, Department of Ecology and Genetics, Uppsala University, Uppsala, Sweden

The manuscript by Boyes and Holland describes an effort to assemble both the nuclear and mitochondrial genome of a male yellow-tailed moth. The procedure includes multiple sequencing techniques allowing for both primary assembly of contigs from long-read libraries (PacBio), polishing with linked reads (10X) and scaffolding with chromosome interaction information (HiC). The highly contiguous (seven very short scaffolds, that only sum up to 0.24 Mb in total, that are not linked to chromosomes) final assembly contains 21 autosomes, the Z-chromosome and the mitochondrial genome. This will be a valuable resource for both population genetic analyses within the species/genus and comparative genomics studies in insects in general and in Lepidoptera in particular. The study sets high standards for the methodology of genome assembly by using and efficiently combining state-of-the-art sequencing technologies and novel bioinformatic pipelines.     

**Minor comments:**

**Abstract:**

1. Maybe omit ‘majority of the’.
2. Information about that the mitochondrial genome is also assembled should be included.
3. Perhaps mention briefly that the number of assembled chromosomes is lower than many previously sequenced lepidopterans – perhaps indicating fusions in the *Euproctis* lineage?

**Introduction:**

1. A brief description of the abundance and distribution range of this species in the AABI area and globally would be very informative.
2. Perhaps it could be of interest to characterize species specific and general biological questions that can be addressed using this genomic resource.

**Genome sequence report + Methods:**

1. The first sentence in these two sections is redundant. Perhaps it is sufficient to describe the sampling location, collector, sample ID and date of sampling (missing) in the methods section and omit the redundant details in the GSR section.

**Genome sequence report:**

1. Omit ‘The majority,”?
2. Add information about that the mitochondrial genome was assembled (including assessment of completeness, e. g. exact length of the scaffold)?
3. The finding that scaffolds are anchored to only 21 autosomes is a bit surprising given that most lepidopterans sequenced so far have 31 chromosome pairs. This could be another example of chromosome rearrangements (here potentially fusions, similar to what has been observed in e.g. *Heliconius melpomene*)? This might be worth mentioning briefly.
4. Were RNAseq sequencing reads used in the downstream analysis?
5. Table 1. Unclear if the “Number of contigs” corresponds to the number of contigs before or after the manual correction of the Hi-C map?

**Methods:**

1. ‘specimen was’ instead of ‘specimens were’.
2. Second paragraph. The sample ID can be given in the first part of the section where sample location, collector, date of sampling etc. is stated.
3. 4 ^th^ line, left column under Figure 1. ‘its concentration RNA assessed’ should perhaps be ‘the RNA concentration assessed’.
4. Information about RNAseq library preparation method, sequencing technique applied and data availability of RNAseq reads is missing.

**Reflection:**

1. An annotation with the RNAseq data generated here in combination with previously available lepidopteran gene models would add useful information for downstream analyses using this resource.

Are sufficient details of methods and materials provided to allow replication by others?

Yes

Is the rationale for creating the dataset(s) clearly described?

Yes

Are the datasets clearly presented in a useable and accessible format?

Partly

Are the protocols appropriate and is the work technically sound?

Yes

Reviewer Expertise:

Comparative genomics, population genetics, molecular evolution.

We confirm that we have read this submission and believe that we have an appropriate level of expertise to confirm that it is of an acceptable scientific standard, however we have significant reservations, as outlined above.

# Peer review: Reviewer response for version 1

**DOI:** 10.21956/wellcomeopenres.18991.r45833

**Published:** 2021-09-13

**Authors:** Steven M. Van Belleghem

**Affiliations:** 1 Department of Biology, University of Puerto Rico-Rio Piedras, San Juan, Puerto Rico

Douglas Boyes and Peter Holland report a high-quality genome assembly for the yellow-tail moth from the UK. They do this using PacBio and 10X sequencing and scaffolded the initially obtained contigs using Hi-C data. The data and assembly seem of high quality and the methods used are rigorous. The assembly contains 30 scaffolds assigned to 22 chromosomes and has a 98.6 BUSCO completeness. I believe this chromosome level assembly will be a valuable tool for future studies.

**Abstract:**

- I suggest using a more precise alternative to the word ‘majority’.

**Introduction:**

- Is it possible to give more precise information on the geographic distribution of this species?

**Results:**

- I do not see any results on the RNA-seq data. Was a transcriptome assembly performed? I also do not see accession numbers to these data in Table 1.
- An assessment of the repetitiveness (TE content) of the genome could be useful.

Are sufficient details of methods and materials provided to allow replication by others?

Yes

Is the rationale for creating the dataset(s) clearly described?

Yes

Are the datasets clearly presented in a useable and accessible format?

Partly

Are the protocols appropriate and is the work technically sound?

Yes

Reviewer Expertise:

Genomics, functional genomics, insects

I confirm that I have read this submission and believe that I have an appropriate level of expertise to confirm that it is of an acceptable scientific standard, however I have significant reservations, as outlined above.
